# Constructing an assessment framework for the quality of asthma smartphone applications

**DOI:** 10.1186/s12911-019-0923-8

**Published:** 2019-10-15

**Authors:** Zhifang Guan, Liu Sun, Qian Xiao, Yanling Wang

**Affiliations:** Capital Medical University, School of Nursing, Beijing, China

**Keywords:** Asthma, Apps, Self-management, Evaluation index, Delphi survey

## Abstract

**Background:**

Enhancing the self-management capability of asthma patients can improve their level of asthma control. Although the use of mobile health technology among asthmatics to facilitate self-management has become a growing area of research, studies of mobile health applications (apps), especially for evaluating indicators of asthma apps, are deficient in scope. This study aimed to develop a reliable framework to assess asthma apps (i.e., content and behavior change strategies) using the Delphi survey technique.

**Methods:**

An initial list of quality rating criteria for asthma apps was derived from reviewing the literature and experts in the fields of respiratory disease and nursing informatics rated the items on the list in three rounds. The weights of items were determined employing an analytic hierarchy process (AHP).

**Results:**

Sixty-two items were retained within 10 domains. Consensus was reached on 32 items concerning asthma self-management education, 25 items concerning behavioral change strategies, and five items concerning principles for app design. There was moderate agreement among participants across all items in round three. The weights of the dimensions, sub-dimensions, and items ranged from 0.049 to 0.203, 0.138 to 1.000, and 0.064 to 1.000, respectively. All random consistency ratio values were less than 0.1.

**Conclusions:**

Asthma self-management education and strategies are essential parts to support self-management for patients. This analysis provides evidence of evaluating criteria for apps targeting chronic and common diseases.

## Background

Asthma is the most common chronic respiratory disease affecting up to 18% of the people in the world [1]. An estimated 334 million people suffer from asthma [2], and the disease is uncontrolled for many patients in developing and undeveloped countries. For example, in Asia, asthma was controlled in only 2.5% of the affected population in 2006 [3], burdening patients, families, governments, and healthcare systems [4]. To prevent the processes of asthma, a myriad of effective measures have been identified and international guidelines concerning asthma self-management education have been promulgated, that have had a positive effect on outcomes [5].

Asthma causes long-term inflammation in the lungs that requires patients to modify their lifestyles—such as smoking cessation and the avoidance of passive smoke. Therefore, healthcare providers at the point of care should be skilled and experienced in asthma self-management education and behavior change strategies to improve the quality-of-life of asthma sufferers [6]. Germane to asthma self-management education, numerous randomized controlled trials have demonstrated positive changes in patient-centered outcomes related to education and behavioral interventions [7]. Nevertheless, many healthcare providers lack training in self-management education and many have little time or motivation to help patients develop those skills [8].

As the use of mobile devices and smartphones becomes more ubiquitous, patients could make full use of applications (apps) on these devices for asthma self-management [8]. Currently, apps on mobile devices can enable patients to monitor and manage the disease, obtain education, and improve health behavior. Communication among users or with practitioners can become more frequent with mHealth apps and mobile technology [9]. Therefore, healthcare providers should assist asthmatics in identifying mHealth smartphone apps that help manage the disease and enable them to provide detailed and personalized feedback to patients at any time [10]. For example, the China Internet Network Information Center (CNNIC) released its annual report on the development of the Internet in China in June 2017, indicating China had 751 million Internet users and 724 million mobile Internet users, an increase of 28.3 million from 2016 [11]. Two hundred fifty-nine thousand mHealth apps were available on major app stores worldwide [12]. These apps have the potential to help a variety of patients improve self-management of their long-term, chronic conditions [13].

Although mHealth apps hold promise and provide advantages for improving health, their quality and suitability for use in clinical practice must be evaluated. Currently, user-based rating systems are provided by the Apple App Store and Google Play (previously Android Market). These rating systems allow users to rank apps from one to five stars in terms of criteria such as usability; however, the validity and reliability of these rating systems and ratings have yet to be reported [14]. As long as the mHealth apps available on these platforms do not make misleading advertising claims and protects the data and identities of the users, they can provide benefit to potential users with chronic diseases [15]. Nevertheless, mHealth apps have rarely adhered to evidence-based principles and peer-reviewed guidelines [16]. For example, Rosser and Eccleston reviewed apps for pain management and report that 86% of the apps indicated no involvement of medical professionals [17]. Moreover, the health information delivered on mHealth apps frequently lacks scientific basis and validity [18]. Furthermore, malfunctions, breaches of patient confidentiality, and conflicts of interests involving apps all conspire against the provision of safe patient care [19]. The staggering number and variety of these mHealth apps makes it difficult for clinicians and the public to identify which of the apps are the safest and most effective [20, 21]. In addition, a lack of standardized rating tools further limits the potential use of apps as part of legitimate healthy lifestyle interventions. Although several assessment frameworks have been published to help rate app quality (e.g., Huckvale et al. developed criteria to assess the content quality of asthma apps [22], and Tinschert et al. applied review frameworks [i.e., behavior change techniques and information] to investigate the potential of asthma apps for self-management [23]), no single instrument addresses the unique combination of information and behavior strategies necessary for asthma patients to effectively self-manage their care.

Clearly, an objective and reliable instrument is necessary to rate the quality of mHealth apps—especially those related to asthma. This instrument initially could be used by researchers and later be made available to app developers and health professionals. This study aimed to develop a reliable and multidimensional index system for rating the mHealth apps for asthma patients that would satisfy the following criteria: (1) provides evidence for patients with asthma and healthcare providers for choosing apps to treat asthma; (2) presents a reference for developers to design asthma apps systematically and scientifically; (3) contributes to improving quality evaluation standards for apps targeting chronic and common diseases.

## Methods

### Study design

To develop a reliable and multidimensional assessment framework for rating the mHealth apps for asthma patients, a three round Delphi survey was conducted using paper-based forms. Experts were asked to indicate the importance of each item based on a 5-point Likert scale from 1 (i.e., *not important*) to 5 (i.e., *extremely important*) [24]. Experts provided feedback between each round of the survey and results were summarized. In a Delphi survey, the multi-round iterative process generally continues until the experts arrive at a common understanding of the qualitative data [25].

No standard methods are available to determine consensus levels [26]. In this study, consensus between participants was measured using the mean importance rating, the coefficient of variation (CV is the ratio of the standard deviation of the responses of the experts on a specific item to its corresponding mean average), and the percentage important (defined as the percentage of respondents who rated a particular item as *extremely important)* [27]. Items were either retained, removed, modified, or added in each Delphi round, based on this standard to reach consensus. The criterion of the mean importance rating and the percentage important is the mean of all items minus their standard deviation, and an item whose score greater than or equal to the criterion is preserved. The criterion of the coefficient of variation is all items’ mean plus standard deviation, keeping the items whose score below or equal to the criterion. When item failed to meet either of the above criteria, were deleted. When the item meets one or two criteria, the decision was made after the discussion of a research group consisting of one associate professor, one university lecturer and three master degree students. Data analysis was performed by two of the authors.

After two rounds of the Delphi survey, the relative importance of each item (e.g., *Asthma is a chronic respiratory disease, together with airway hyperresponsiveness and airway inflammation,* and *Asthma cannot be cured, but can be effectively controlled through effective management*) was calculated. The analytic hierarchy process (AHP) fundamental scale developed by Saaty for pairwise comparisons was then used to construct the judgment matrix to calculate the weight of each item [28].

**Fig. 1:**
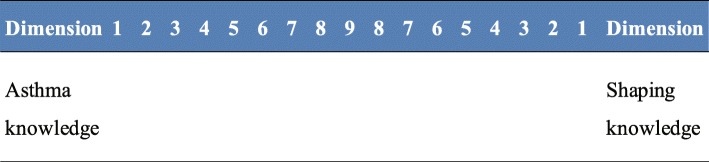
A sample question of pairwise comparisons in the questionnaire. 1: Equal importance; 3: Moderate importance of one over another; 5: Essential or strong importance; 7: Very strong importance; 9: Extreme importance; 2, 4, 6, 8: Intermediate values between the two adjacent judgements

In the study, each participant compared all criteria pairwise with each other using a scale ranging from 1 to 9 to 1. For each pair, participants had to select which was more important, see Fig. 1. After collecting the questionnaires, the AHP module matrix written with Excel was utilized for data analysis.

In addition, a consistency test was conducted of the judgment matrix. When the random consistency ratio (CR) was less than 0.1, then the judgment matrices were considered acceptable.

The CR coefficient is calculated as follows [29].

CI represents the consistency index, and RCI represents random consistency index, which was used to modify the CI value (if n > 2). n means the order of the matrix.

1 $$ \mathrm{CI}=\left({\uplambda}_{\mathrm{max}}-\mathrm{n}\right)/\left(\mathrm{n}-1\right) $$

λ_max_ means an approximation of the maximum eigenvalue of the judgement matrix.

The CR coefficient is obtained by dividing the CI value by RCI. The value of RCI of the reciprocal matrix of 1–9 orders is given in Table 1.

2 $$ \mathrm{CR}=\mathrm{CI}/\mathrm{RCI}\ (2) $$

**Table 1:** The value of random consistency index (RCI) of the reciprocal matrix of 1–9 orders

Matrix order	1	2	3	4	5	6	7	8	9
RCI	0	0	0.58	0.90	1.12	1.24	1.32	1.41	1.45

### Participant recruitment

Participants were active in the field of respiratory disease having expertise in asthma management. Experts were identified from Beijing and Tianjin working in general hospital or medical university. Experts were identified according to the following criteria: (1) they had to be engaged in the field of respiratory for more than 5 years; (2) they had to hold intermediate professional titles and a college degree or graduate degree in a respiratory field; (3) they had to be willing take part in all rounds of the Delphi survey.

### Procedure

#### Developing the initial index system

The asthma self-management education and behavior change techniques (BCT) and corresponding items to be evaluated through the Delphi survey were identified through (1) a content analysis of 110 asthma apps from the Apple App Store and Google Play that covered self-management education and functions [30] and (2) a review of the relevant literature.

For the literature review, major databases (i.e., PubMed, Ovid, EBSCO, Elsevier, SpringerLink, SinoMed, China National Knowledge Infrastructure [CNKI], and WanFang) were searched using the keywords *asthma* and *self-management* or *behavior change techniques* or mobile app* and evaluate* or mobile app* and assess* between the date January 2013 and October 2017. There were 10,545 articles retrieved, 6363 were removed as duplicates, and after initial screening of title and abstract, only 734 articles that reported asthma self-management education, behavior change techniques and evaluation instrument of apps were included. After reading their full text, 14 major relevant articles were identified.

App assessment items were extracted by analyzing the major relevant literature [2, 6, 9, 23, 31–40] by two authors, who then drafted a set of provisional dimensions for the items and sorted them by dimension. A total of 105 items were identified. That fell into 10 major dimensions: (1) goals and planning, (2) feedback and monitoring, (3) shaping knowledge, (4) social support, (5) reward and threat, (6) natural consequences, (7) improving the compliance, (8) asthma information, (9) patient skills training, and (10) non-pharmacological interventions. After removing redundant items, 87 items remained, which the authors then grouped into 23 sub-dimensions, defining the 10 major dimensions. They were (1) goal setting (outcome/behavior), (2) asthma action plans, (3) self-monitoring of behavior, (4) self-monitoring of outcomes of behavior, (5) feedback, (6) demonstration of the behavior, (7) behavior substitution, (8) practical social support, (9) emotional support, (10) social reward, (11) threat, (12) information about health consequences, (13) salience of consequences, (14) prompts. (15) regulation, (16) the nature of asthma, (17) asthma medication, (18) management of asthma exacerbation, (19) management of comorbidities, (20) peak flow meter usage, (21) inhaler technique, (22) identifying and avoiding risk factors, (23) good life style. The research group ensure that the survey questionnaire did not include items that were difficult to understand or repetitive. The preliminary list of proposed items underwent a process of revision and adaptation to reach a definitive version that was approved by all authors. The questionnaire was sent to each expert who agreed to participate in the study and the Delphi process was explained to these participants. The original list appears in Table 2.

**Table 2:** The items in the questionnaires of round 1 Delphi survey

Dimensions	
	1. Goals and planning
	2. Feedback and monitoring
	3. Shaping knowledge
	4. Social support
	5. Reward and threat
	6. Natural consequences
	7. Improving the compliance
	8. Asthma information
	9. Patient skills training
	10. Non-pharmacological interventions
Sub- dimensions	1.1 Goal setting (outcome/behavior)
	1.2 Asthma action plans
	2.1 Self-monitoring of behavior
	2.2 Self-monitoring of outcomes of behavior
	2.3 Feedback
	3.1 Demonstration of the behavior
	3.2 Behavior substitution
	4.1 Practical social support
	4.2 Emotional support
	5.1 Social reward
	5.2 Threat
	6.1 Information about health consequences
	6.2 Salience of consequences
	7.1 Prompts
	7.2 Regulation
	8.1 The nature of asthma
	8.2 Asthma medication
	8.3 Management of asthma exacerbation
	8.4 Management of comorbidities
	9.1 Peak flow meter usage
	9.2 Inhaler technique
	10.1 Identifying and avoiding risk factors
	10.2 Good life style
Items	1.1.1 Ask patients to reflect on what they would consider as good asthma control
	1.1.2 The purpose of asthma control is to have good asthma control and no limited activities
	1.1.3 Set specific behavior goals in terms of the behavior to be achieved
	1.1.4 State the factors that influence the behavior, and generate strategies that overcome barriers and increase facilitators
	1.1.5 Set goals in terms of a positive outcome
	1.1.6 Re-set new goals in light of achievement
	1.2.1 Patient should be provided with an asthma action plan, and updated in time
	1.2.2 State that asthma action plan helps patient to recognize and response appropriately to worsening asthma
	1.2.3 Set detailed planning of performance of the behavior
	1.2.4 Patients need to affirm commitment to change the behavior
	2.1.1 States that the valid approaches for self-monitoring are PEF monitoring and symptom recognition
	2.1.2 Provides reminders to monitor PEF everyday, and can record details
	2.1.3 Provides reminders to monitor symptoms everyday, and can record details
	2.1.4 Provides a diary to record medication
	2.1.5 Provides a diary to record patients’ feeling
	2.1.5 Provides a diary to record return visit
	2.2.1 Provides a diary to record lung function test
	2.2.2 Provides a diary to record worsening asthma-related events
	2.2.3 Provides a diary to record factors related to worsening asthma, such as weather
	2.2.4 Provides asthma assessment tools
	2.3.1 Monitors and provides informative or evaluative feedback on performance of the behavior
	2.3.2 Provide professional feedback information based on patients’ inhaler technique
	2.3.3 Provides evaluative feedback on asthma status
	2.3.4 Provides informative feedback on asthma severity based on PEF values
	2.3.5 Generates PEF/symptoms summary visualization
	2.3.6 Provides result feedback through connecting medical devices
	3.1.1 Provide video tutorials or animations of peak flow meter use
	3.1.2 Provide video tutorials or animations of inhaler devices use
	3.1.3 Provides video tutorials or animations to display instructions of spacer for patients
	3.2.1 States wanted or neutral behavior to substitute the unwanted behavior
	3.2.2 States repetition of the wanted behavior
	4.1.1 Allows establishing a cooperative relationship between doctors and patients
	4.1.2 Allows setting goals by patients and doctors
	4.1.3 Provides video tutorials or animations about asthma information introduced by medical workers
	4.1.4 Allows users to share health data with medical workers through email
	4.1.5 Allows patients to communicate with patients with controlled asthma
	4.2.1 States that friends, relatives and medical workers should provide emotional support
	4.2.2 Provides encouragement and consultation from friends, relatives and medical workers
	4.2.3 Provides self-incentive in performing the behavior
	5.1.1 Send incentive information if there has been progress in performing the behavior
	5.2.1 Do not send incentive information if patients with unwanted behavior
	6.1.1 Provides information about health consequences of performing the behavior
	6.1.2 Provides information about social and environmental consequences of performing the behavior
	6.2.1 Provides methods specifically designed to emphasize the consequences of performing the behavior
	7.1.1 Set environmental or social stimulus in order to prompt the behavior
	7.1.2 Allows users to set reminder for medication/return visit
	7.1.3 Provides reminders for checking inhaler to ensure inhalers are not empty
	7.1.4 Provides reminders for checking inhaler to ensure inhalers are in date
	7.2.1 Provides stress-reduction strategies to prevent symptoms from worsening
	7.2.2 States the importance of avoiding use of multiple different inhaler types
	8.1.1 Asthma is a chronic respiratory disease, together with airway hyperresponsiveness and airway inflammation
	8.1.2 Asthma is caused by a combination of endogenous (genetic) and external (environment) causes.
	8.1.3 Respiratory symptoms of asthma are wheeze, shortness of breath, chest tightness and cough
	8.1.4 Asthma severity can be assessed as mild asthma, moderate asthma, and severe asthma
	8.1.5 Asthma cannot be cured, but can be effectively controlled through effective management.
	8.1.6 Early controller treatment of asthma is critical to achieving optimal outcomes
	8.2.1 Asthma medications include controller medications and reliever medications
	8.2.2 Controller medications can be used to reduce airway inflammation, control symptoms, and reduce future risks
	8.2.3 Controller medications should be used for regular
	8.2.4 Reliever medications are used to relief breakthrough symptoms
	8.2.5 Reliever medications are used as needed
	8.2.6 Local side-effects of ICS include oral thrush and dysphonia
	8.2.7 Side-effects of oral corticosteroids include osteoporosis, hypertension, and diabetes, etc.
	8.2.8 Side-effects of ß_2_-agoinsts include tachycardia and tremor
	8.2.9 Patients need to carry asthma reliever medications (such as Ventolin solution) with them in case of emergency
	8.3.1 Early signs and symptoms of worsening asthma are sneezing, runny nose, dry cough, shortness of breath, and chest tightness, etc.
	8.3.2 Symptoms of asthma exacerbations are a progressive increase in symptoms of shortness of breath, cough, wheezing or chest tightness
	8.3.3 It is important to adjust treatment plan and went to see the doctor in time
	8.3.4 Patients were removed from the allergen environment, inhale ß_2_ agonist, and went to see the doctor in time
	8.4.1 Complications should be treated, such as rhinitis, sinusitis, and symptomatic gastroesophageal reflux disease
	8.4.2 Obese patient should lose weight
	9.1.1 Operational criteria of peak expiratory flow meter: taking a deep breath; sealing your mouth tightly around the mouthpiece; blowing as hard and as fast as you soon; checking the number, re-setting the pointer to zero; and repeating two more times
	9.1.2 A peak flow meter is used for monitoring lung function changes in patients
	9.1.3 Patients should use the same meter each time
	9.2.1 Patients should be encouraged to participate in the choice of inhaler device
	9.2.2 Emphasizes the importance of correct inhaler technique
	9.2.3 States that patients should breathe deeply and hold their breath for a few seconds for effective use of inhaler devices
	9.2.4 Patients should rinse and spit the mouse after using the inhaled hormone
	9.2.5 States that appropriate use of spacer device can improve effect and reduce adverse drug reactions
	10.1.1 States identifying risk factors that make asthma worse
	10.1.2 States the importance of avoidance of environmental smoke exposure
	10.1.3 States the importance of avoidance of occupational exposures
	10.1.4 States the importance of avoidance of medications that may make asthma worse
	10.1.5 States the importance of avoidance of allergens exposure
	10.2.1 States the importance of consuming a diet high in fruit and vegetables
	10.2.2 States the importance of avoidance of indoor air pollutants
	10.2.3 States the importance of engaging in regular physical activity

#### Round 1 of Delphi survey

In round one of the Delphi survey, in November, 2017, a total of 25 experts agreed to participate in the Delphi survey. They represented six hospitals and/or academic institutions in Beijing and Tianjin, including Capital Medical University School of Nursing, Beijing Chaoyang Hospital affiliated to Capital Medical University, Xuanwu Hospital affiliated to Capital Medical University, Beijing Children’s Hospital affiliated to Capital Medical University, China-Japan Friendship Hospital in Beijing, and Tianjin Medical University General Hospital. All expert participants in round one were female whose ages ranging from 31 to 55 years (mean = 42.28; SD = 6.58). Participants were drawn from three main occupational groups: nurse educators in higher education, clinical head nurses, and respiratory physicians.

The first-round questionnaire contained 10 dimensions, 23 sub-dimensions, and 87 items. In addition, the questionnaire contained of 50 items related to behavioral change strategies and 37 items related to asthma self-management education.

The first section of the first-round questionnaire (1) describes the background and objectives of the study and (2) specifies the deadline for returning the completed questionnaire. The second section elicits the opinions of experts concerning not only the revision, addition, and/or deletion of any items, but also the importance of each item based on a 5-point Likert scale. In addition, participants were given an option to suggest additional items. The third section elicited demographic information from the participants, which included professional background (i.e., years engaged in work, educational background, professional title, and affiliation). In this section, the expert degree of authority also was measured. The authority coefficient (C_r_), in relation to the participants’ technical ability to evaluate the items, was determined by two factors: the participants’ familiarity with the items (C_s_) and the judgment criteria for the items (C_a_) [41]. Familiarity with items was measured on a 5-point Likert Scale in the following order and score: unfamiliar (0), somewhat unfamiliar (0.2), somewhat familiar (0.5), very familiar (0.8), extremely familiar (1). The judgment criteria for the items encompassed parameters such as experience in asthma self-management, theoretical analysis of items, knowledge of the literature, and instinct. A scoring system was used to rate the experts’ criterion for their judgments (see Table 3) [42], and the rating was done by the participants. Informed consent was obtained from each participant once they accepted the invitation to participate.

**Table 3:** Criterion for judgment and scoring system

Judgment Criterion	The Degree of Impact on Experts’ Judgement		
	Large	Medium	Small
	Impact	Impact	Impact
Experience	0.5	0.4	0.3
Theoretical analysis	0.3	0.2	0.1
Knowledge of literature	0.1	0.08	0.05
Instinct	0.1	0.07	0.05

#### Round 2 of Delphi survey—determining the weight of each item through AHP

**Fig. 2:**
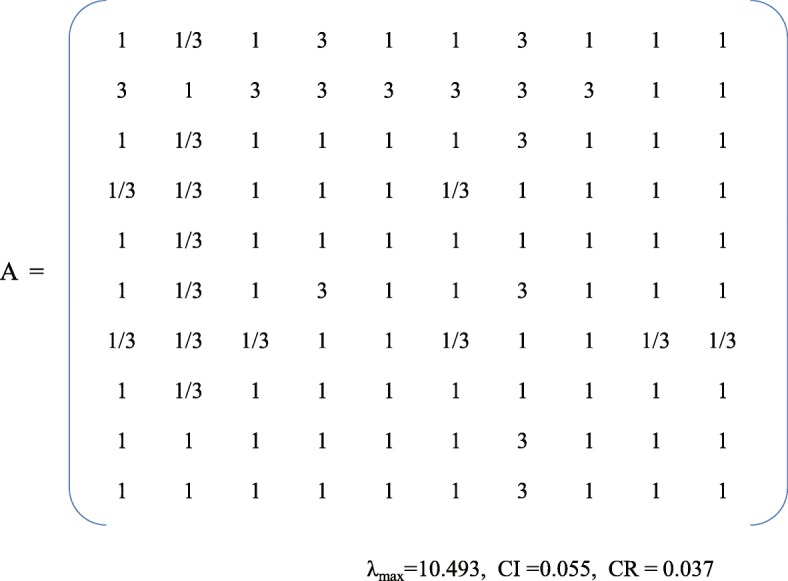
Pairwise comparison matrix A for 10 dimensions

**Fig. 3:**
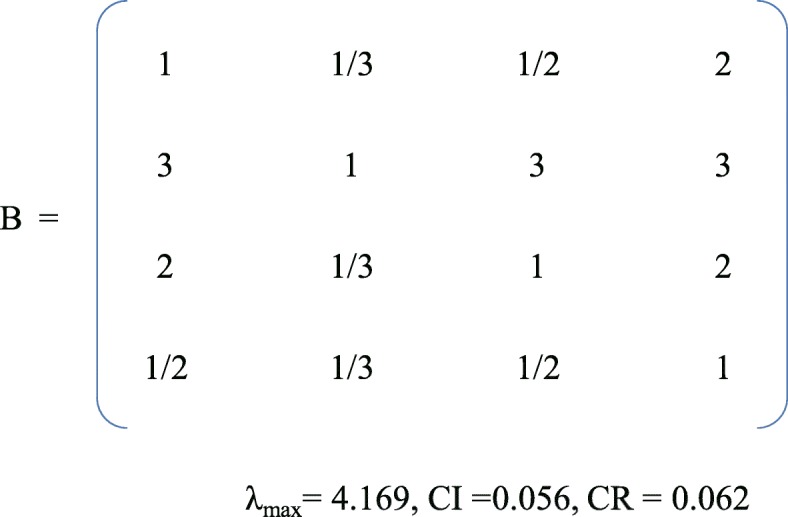
Pairwise comparison matrix B for sub-dimension of asthma knowledge

The second round of the Delphi survey ended in January, 2018 with 24 experts participating. Of these, 20 participated in the first-round and four new experts were added. The five participants who dropped out after the first round did so because of vacations. The second-round questionnaires were based on the results of the first-round, according to both the agreement on each item and the suggestions of experts. Participants were required to (1) re-rate the importance of the items on the questionnaire regarding the apps and (2) provide additional edits, revisions, suggestions, comments, and/or questions. The three sections of the round two questionnaire followed the same format as the round one questionnaire. However, in the second-round questionnaire, expert participants were provided judgment criteria to evaluate the relative importance of 10 dimensions, using a series of pairwise comparisons, and the median of the score of each item was used to construct judgement matrices of the 10 dimensions by first author (see Fig. 2) [43]. Meanwhile, the number of sub-dimension and items are large, affects the judgement of experts. So, in this study, the average score of importance of each item minus the average score of other items from the second Delphi round was used to extract the intensity of importance (formula 3), then construct judgment matrices, Table 4 exhibits standard of pairwise comparison values for sub-dimensions and items [44]. According to formula 3 and standard of intensity of importance, we got the judgement matrices B sub-dimension of asthma knowledge (see Fig. 3).

3 $$ \mathrm{B}={\left({\mathrm{b}}_{\mathrm{i}\mathrm{j}}\right)}_{\mathrm{n}\upchi \mathrm{n}}\ \left({\mathrm{b}}_{\mathrm{i}\mathrm{j}}={\mathrm{b}}_{\mathrm{i}}-{\mathrm{b}}_{\mathrm{j}},\mathrm{i},\mathrm{j}=1,2,\dots, \mathrm{n}\right) $$

**Table 4:** Standard of pairwise comparison values for sub-dimensions and items

	Definition	Intensity of importance
0.25<A_ij_–A_ik_ ≤ 0.50	A_ij_ is moderately more important than A_ik_	3
0.75<A_ij_–A_ik_ ≤ 1.00	A_ij_ is strongly more important than A_ik_	5
1.25<A_ij_–A_ik_ ≤ 1.50	A_ij_ is very strongly more important than A_ik_	7
1.75<A_ij_–A_ik_	A_ij_ is extremely more important than A_ik_	9
Intermediate value between the two adjacent judgements		2,4,6,8

The eigenvector of judgement matrix was calculated, and then the weight of each item was obtained.

#### Round 3 of Delphi survey

The third round of the Delphi survey ended in April, 2018. Eleven participants from the first round were invited and agreed to take part. Their ages ranging from 32 to 53 (mean = 41.00; SD = 6.55) in round three. Table 5 exhibits the demographic data and characteristics of the expert participants who took part in the three rounds of the Delphi survey. The round-three questionnaires featured the format as the round-one and round-two questionnaires. The expert participants were asked to re-rate the importance of the items on the questionnaire, using the same 5-point Likert scale.

**Table 5:** Demographic data and characteristics of the expert panel

	Round 1 (*N* = 25) N (%)	Round 2 (*N* = 24) N (%)	Round 3 (*N* = 11) N (%)
Age (years)			
< 40 years old	11 (44.0)	11 (45.8)	6 (54.5)
40–50 years old	10 (40.0)	9 (37.5)	3 (27.3)
> 50 years old	4 (16.0)	4 (16.7)	2 (18.2)
Work experience (years)			
10 years or less	12 (48.0)	12 (50.0)	7 (63.6)
10–20 years	10 (40.0)	9 (37.5)	2 (18.2)
More than 20 years	3 (12.0)	3 (12.5)	2 (18.2)
Education			
Bachelor’s degree	5 (20.0)	5 (20.8)	1 (9.1)
Master’s degree	13 (52.0)	10 (41.7)	7 (63.6)
PhD	7 (28.0)	9 (37.5)	3 (27.3)
Professional Title			
Intermediate title	11 (44.0)	8 (33.3)	4 (36.4)
Senior vice title	10 (40.0)	11 (45.8)	5 (45.4)
Senior title	4 (16.0)	5 (20.8)	2 (18.2)
Affiliation			
Educational institution	6 (24.0)	5 (20.8)	5 (45.5)
Clinical practice	19 (76.0)	19 (79.2)	6 (54.5)

Participants remained anonymous to each other during the entire survey process, and they were required to complete the questionnaires within 3 weeks. Data collection was performed by the same member of the research team. All of the questionnaires and the data collection procedures were checked by the all members of the research team to assure credibility. The data was double-entered and checked for accuracy.

### Data analysis

Quantitative data were entered into Microsoft Excel 2010 and IBM SPSS 20.0 Statistics for Windows for analysis, and descriptive statistics were used. The rating for each item was analyzed and expressed as a mean value with standard deviation (SD). Following this, non-parametric statistics (e.g., chi-squared test of association) were used to determine the possibility of any response group bias. Coefficient of variation (CV) and Kendall’s coefficient of concordance (Kendall’s *W*) were used to test the dispersion of the participants’ opinions. A *p* value of less than 0.05 was considered statistically significant.

## Results

### Survey results

In round one of the Delphi survey, the authoritative coefficient for the expert participants ranged from 0.80 to 0.96, with an average authority coefficient of 0.89. The mean importance ratings for dimensions ranged from 3.28 to 4.88, and the coefficient of variation ranged from 0.07 to 0.37. The mean importance ratings for sub-dimensions ranged from 3.20 to 4.86, and the coefficient of variation ranged from 0.04 to 0.39. The mean importance ratings for items ranged from 3.44 to 4.92, and the coefficient of variation ranged from 0.06 to 0.32. In round two, the participants’ degree of authority ranged from 0.65 to 1.00, with an average authority coefficient of 0.91. The mean importance ratings for dimensions ranged from 4.00 to 4.92, and the coefficient of variation ranged from 0.06 to 0.23. The mean importance ratings for sub-dimensions ranged from 4.00 to 4.88, and the coefficient of variation ranged from 0.07 to 0.26. The mean importance ratings for items ranged from 3.54 to 4.83, and the coefficient of variation ranged from 0.08 to 0.34. In round three, the participants’ degree of authority ranged from 0.67 to 0.98, with an average authority coefficient of 0.90. The mean importance ratings for the dimensions ranged from 4.36 to 4.91, and the coefficient of variation ranged from 0.06 to 0.20. The mean importance ratings for the sub-dimensions ranged from 4.27 to 4.91, and the coefficient of variation ranged from 0.06 to 0.18. The mean importance ratings for the items ranged from 4.45 to 4.91, and the coefficient of variation ranged from 0.06 to 0.19. After modification of the items in the questionnaire, the coordination results in the third round were acceptable—the Kendall’s *W* ranged from 0.654 to 0.693 (see Table 6).

**Table 6:** The concordance degree of the expert’s opinions

	Items	Kendall’s *W*	χ^2^	*P*
Round 1	Dimensions	0.440	105.692	< 0.001
	Sub-Dimensions	0.410	226.335	< 0.001
	Items	0.412	859.754	< 0.001
Round 2	Dimensions	0.569	104.780	< 0.001
	Sub-Dimensions	0.548	239.506	< 0.001
	Items	0.507	758.071	< 0.001
Round 3	Dimensions	0.693	69.335	< 0.001
	Sub-Dimensions	0.654	150.503	< 0.001
	Items	0.656	413.448	< 0.001

### Item modifications

Table 7 illustrates the requirements for consensus for all items in rounds 1, 2 and 3. Criteria reaching consensus were retained while those not reaching consensus were removed.

**Table 7:** Requirements for consensus in rounds 1, 2 and 3

Criterion	Round 1	Round 2	Round 3
Percentage important	≥42%	≥44%	≥65%
Mean importance rating	≥4.09	≥4.21	≥4.53
Coefficient of variation	<0.23	<0.20	<0.16

In round one of the Delphi survey, based on consensus criteria and team discussion, two dimensions were deleted, *reward and threat* was deleted because of its negative impact on patients, and *natural consequences* were deleted because of perceived duplication. Five of sub-dimensions (i.e., *social reward*, *threat*, *information about health consequences*, *salience of consequences*, and *regulation*) and 24 of items were deleted. An example for retaining items based on consensus criteria was shown in Table 8. In addition, the three dimensions were changed, *improving the compliance* was reworded as *prompts*, *Asthma information* was changed to *asthma knowledge*, and *patient skills training* was reworded as *skills training for effective self-management* because of its inaccurate language. Seven of sub-dimensions (i.e., *prompts, the nature of asthma, management of comorbidities, peak flow meter usage, inhaler technique, identifying and avoiding risk factors, good life style*) and 42 of items were changed. Additionally, two new dimensions (i.e., *ease of use* and *usability*), eight new sub-dimensions (*warnings, accessibility, automation, unconstraint, user-friendly interface, security, usefulness of knowledge, rate of update*), and 15 new items were proposed by the participants to be added to the questionnaire after the first-round survey, which resulted in the inclusion of 10 dimensions, 25 sub-dimensions, and 73 items in the second-round questionnaire. Moreover, the wording of most items was revised based on the expert panel’s comments and re-ordered the items concerning asthma self-management education and behavior change strategies.

**Table 8:** An example for retaining items based on the requirements for consensus in round one

Dimension	Mean	CV	The percentage important	Decision
Asthma knowledge	4.80	0.083	80%	Remaining
Reward and threat	3.28	0.371	20%	Removed
Social support	4.08	0.229	40%	Remaining after discussion
Criterion	≥4.09	<0.23	≥42%	

In round two, no new items were generated. Based on the criteria and team discussion, three of sub-dimensions (i.e., *behavior substitution*, *unconstraint*, and *security*) and 10 items were deleted. Three items were changed (*Information released by apps can help patients to make decision* was changed to *The app can be easily accessed and obtained information.*; *The app can help patients to improve the efficiency of self-management* was changed to *Information released by apps is to patients’ needs and value; The app can help patients to know the recent knowledge* was changed to *The app is updated regularly and timely*) based on the suggestions of the expert participants. As a result, 10 dimensions, 23 sub-dimensions, and 63 items were generated for the second round of the Delphi survey. In addition, we added descriptions and/or examples for items in the questionnaire.

In round three, only one item (i.e., *the app allows users to re-set the goals based on patients’ health data*) was deleted because difficult to be measured. As a result, the final version of asthma apps assessment framework comprised 10 dimensions, 23 sub-dimensions, and 62 items after three Delphi surveys. See Table 9.

**Table 9:** Asthma apps assessment framework and weight value of each item after three-round Delphi survey

Dimensions	Weight	Sub-Dimensions	Weight	Items	Criteria/Example	Weight	Overall Weight
The following knowledge is included in the apps (i.e., presented in words, pictures, video, etc.):							
Asthma knowledge	0.105	Basic fact about asthma	0.182	Definition of asthma	Asthma is a chronic respiratory disease, together with airway hyperresponsiveness and airway inflammation	0.086	0.0016
				Causes of asthma	Asthma is caused by a combination of endogenous (genetic) and external (environment) causes	0.123	0.0024
				Respiratory symptoms of asthma	Repeated episodes of wheeze, shortness of breath, chest tightness and cough	0.177	0.0034
				Prognosis of asthma	Asthma cannot be cured, but can be effectively controlled through effective management	0.253	0.0048
				Early treatment	Early controller treatment of asthma is critical to achieving optimal outcomes	0.361	0.0069
		Asthma medications	0.439	Categories of asthma medications	Asthma medications include controller medications and reliever medications	0.138	0.0063
				Roles and usage of controller medications	Controller medications can be used to reduce airway inflammation, control symptoms, and reduce future risks, which must be used regularly	0.240	0.0110
				Roles and usage of reliever medications	Reliever medications can be used to relief breakthrough symptoms, which must be used as needed	0.182	0.0084
				Side-effects of asthma medications	Local side effects of ICS include oral thrush and dysphonia; osteoporosis, hypertension, and diabetes, etc. in high dose steroids; tachycardia and tremor in ß_2_-agoinsts	0.096	0.0044
				Carrying reliever medications	Patients need to carry asthma reliever medications (such as Ventolin solution) with them in case of emergency	0.344	0.0158
		Management of asthma exacerbations	0.241	Early signs and symptoms of worsening asthma	The app describes early signs and symptoms of worsening asthma (sneezing, runny nose, dry cough, shortness of breath, and chest tightness, etc.)	0.138	0.0035
				Symptoms of asthma exacerbations	The app describes symptoms of asthma exacerbations (a progressive increase in symptoms of shortness of breath, cough, wheezing or chest tightness)	0.195	0.0049
				Management of asthma exacerbations	For example, patients were removed from the allergen environment, inhale ß_2_ agonist, and went to see the doctor in time	0.391	0.0098
				Management after asthma exacerbations	Seek the cause of acute attack actively, check medication compliance, and adjust treatment plan	0.276	0.0070
		Asthma with comorbidities and triggers	0.138	Comorbidities of asthma	The app describes comorbidities of asthma, such as, rhinitis, sinusitis, and symptomatic gastroesophageal reflux disease, etc.	0.249	0.0036
				Management of comorbidities	The app provides details of treatment of rhinitis, sinusitis, and symptomatic gastroesophageal reflux disease, and psychological intervention, etc.	0.157	0.0023
				Triggers of asthma	The app describes triggers of asthma, such as, occupational factors, environmental factors, weather changes, drug and sports	0.594	0.0086
Skills training for effective self-management	0.203	Peak flow meter use and monitoring	0.667	The purpose of using peak flow meter	A peak flow meter is used for monitoring lung function changes in patients	0.195	0.0264
				Operational criteria for peak flow meter	Take a deep breath, seal your mouth tightly around the mouthpiece and then blow as hard and as fast as you soon. Check the number, re-set the pointer to zero, and repeat two more times	0.391	0.0527
				The same peak flow meter	The patient should use the same peak flow meter each time	0.276	0.0373
				The best time to use peak flow meter	PEF is measured in the morning, and then in the evening (after 10-12 h of the first time)	0.138	0.0186
		Inhaler devices use	0.333	Common inhaler devices	The app describes common inhaler devices, such as pressurized metered dose inhalers (pMDI), pMDI +spacer and dry power inhalers (DPIs)	0.140	0.0094
				The importance of correct inhaler technique	Correct inhaler technique can enhance the medication into lung, reduce asthma attack, and obtain the best clinical effect	0.528	0.0356
				Operational criteria for different inhaler devices	For example, usage of Diskus is that remove mouthpiece cover, position inhaler mouthpiece in mouth and seal lips, inward breath steady and deeply, remove inhaler, hold breath for a few seconds, and rinse mouth	0.332	0.0225
Non- pharmacological strategies	0.094	Measures to treat asthma triggers	0.667	Identifying risk factors that make asthma worse	The app describes factors that make asthma worse, such as allergens exposure, physical and chemical irritants, psychosocial factors, etc.	0.160	0.0100
				Avoidance of environmental smoke exposure	The app provides advice about avoidance of active smoking and passive smoking	0.106	0.0066
				Avoidance of occupation exposures	The app provides advice about avoidance of plant dust, animal dust, etc.	0.255	0.0159
				Avoidance of medications that may make asthma worse	The app provides advice about avoidance of aspirin, NSAIDs, and ß-blockers, etc.	0.255	0.0159
				Avoidance of allergen exposure	The app provides advice about avoidance of domestic mites, furred animals, fungi, and pollen, etc.	0.160	0.0100
				Avoidance of indoor and outdoor air pollution	The app provides advice about avoidance of domestic coal burning, cooking, and traffic pollution, etc.	0.064	0.0040
		Lifestyles	0.333	Avoidance of emotional stress	The app provides advice about relieving emotional stress and encouraging breathing exercises, etc.	0.667	0.0209
				Regular moderate physical activity	Patients should exercise regularly and given appropriate exercise advice	0.333	0.0104
The following behavioral change strategies are employed in apps:							
Goals and planning	0.068	Goal setting (outcome/behavior)	0.667	Allow users to set behavior goals or provide behavior goals	The app allows users to record symptoms and PEF values daily, and assess asthma control level periodically, etc.	0.667	0.0300
				Allows users to set outcome goals or provide outcome goals	The app allows users to set the goals of asthma control	0.333	0.0150
		Action plans	0.333	Explain the purpose of an asthma action plan	An asthma action plan helps patients to identify early symptoms of asthma attacks and respond appropriately to improve asthma control	0.500	0.0113
				Allow making individualized asthma action plan, and updated in time	The app allows doctors to program asthma action plan directly into their phone or users type in manually	0.500	0.0113
Feedback and monitoring	0.084	Feedback	0.250	Provide result feedback information based on patients’ health data	The app can provide advice based on changing PEF, symptoms or ACT scores	0.185	0.0039
				Provide professional feedback information based on patients’ inhaler technique	The app allows users to upload the patients’ inhaler technique video through user-end, and then clinicians check inhaler technique in order to identify problematic steps	0.245	0.0051
				Provide feedback information based on patients’ changing asthma status	The app can provide feedback about severity of asthma based on symptoms or PEF, etc.	0.323	0.0068
				Allow storing and summarizing patients’ recent health data, and generating summary visualization automatically	The app allows storing patients’ data, such as symptoms, PEF or medicine use	0.141	0.0030
				Allow connecting medical devices or wearables to upload data and provide feedback information to patients	For example, after the sensor collecting the patients’ vital signs, the app can send the data to end-users and judge whether the patients’ health is in the normal range	0.106	0.0022
		Self-monitoring of behavior	0.500	Provide a diary to record PEF readings and predicted PEFR will be calculated automatically	The app allows users to type in manually or supports pair to the patients’ Bluetooth device automatically for data exchange	0.195	0.0082
				Provide a diary to record patients’ symptoms.	The app provides a diary to record details about wheezing/shortness of breath/sleep, etc.	0.391	0.0164
				Provide a diary to record medication.	The app provides a diary to record details about medication use, such as categories and frequency, etc.	0.276	0.0116
				Provide a diary to record return visit.	The app provides a diary to record details about return visit, such as frequency and results, etc.	0.138	0.0058
		Self-monitoring of outcomes of behavior	0.250	Provide a diary to record lung function test.	The app provides a diary to record details about lung function test, such as FEV_1_, FVC, etc.		0.0065
				Provide a diary to record worsening asthma-related events.	The app provides a diary to record details about worsening asthma-related events, such as attack symptoms, duration of symptoms, and complications, etc.	0.196	0.0041
				Provide asthma assessment tools	The app provides asthma assessment tools to assess patient progress, such as Asthma Control Test (ACT)	0.493	0.0104
Shaping knowledge	0.105	Demonstration of behavior	1.000	Provide video tutorials or animations of peak flow meter use	The app provides video tutorials or animations to display instructions of peak flow meter for patients	0.500	0.0524
				Provide video tutorials or animations of inhaler devices use	The app provides video tutorials or animations to display instructions of inhaler device for patients	0.500	0.0524
Social support	0.049	Practical support	0.667	Allow establishing a cooperative relationship between doctors and patients, and providing patient-doctor communication platform or interactive consultation service	For example, the app offers online consulting service	0.100	0.0324
		Emotional support	0.333	Provide functions of interactive communication among patients	For example, the app provides functions of sharing information and comment, etc.	0.100	0.0162
Prompts	0.084	Reminder	0.333	Allow users to set reminders for asthma tests.	The app provides details of asthma tests reminder	0.139	0.0039
				Allow users to set medication reminder	The app provides details of medication reminder, such as medication name and dosage, etc.	0.393	0.0110
				Allow users to set reminders for return visit	The app can send information regularly to remind return visit	0.234	0.0066
				Provide reminders for checking inhalers	The app provides reminders for checking the date and medications dosage of inhalers	0.234	0.0066
		Warnings	0.667	Provide alert based on patients’ changing health data	The app can send warning information automatically when there is abnormal data	1.000	0.0561
The following design principles are implemented in the apps:							
Ease of use	0.105	Accessibility	0.429	The app can be easily accessed and obtained information	The app and its contents are accessible to all users (including all kinds of users with access barriers, such as visual impairment, hearing impairment, etc.)	1.000	0.0449
		Automation	0.429	The app can retrieve patients’ data automatically	The app can connect to health apparatuses to improve efficiency of data collection	1.000	0.0449
		User-friendly interface	0.142	All components/screens, menu labels/icons of apps are clear, intuitive, and able to use immediately	Interface design (including menu, background, colors, fonts, etc.) is scientific and reasonable. The operation steps are simple and can be operated according to window prompts without user guide. Navigation is logical and intuitive, and internal and external links are valid	1.000	0.0150
Usability	0.105	Usefulness of knowledge	0.250	Information released by apps is for patient’s needs and value	Information contained within apps is accurate and comprehensive, with high utilization rate	1.000	0.0262
		Rate of update	0.750	The app is updated regularly and timely	The app (including its contents, functions and technology) is updated regularly and timely	1.000	0.0786

### Calculating the weight of items through AHP

In round two, the weights of the dimensions were 0.105, 0.203, 0.094, 0.068, 0.084, 0.105, 0.049, 0.084, 0.105, 0.105, respectively, with a CR of 0.037. The overall weights of the sub-dimensions ranged from 0.015 to 0.135, with CR values from 0 to 0.062. Moreover, the overall weights of the items ranged from 0.002 to 0.079, with CR values ranging from 0 to 0.046.

## Discussion

Much of the literature concerning the evaluation of mHealth apps has merely addressed the technical aspects of apps [45–49]. The purpose of this study was to develop a framework to assess and improve the quality of asthma smartphone apps for use on smartphones. The three-round Delphi survey process produced consensus on the items comprising a framework for assessing the quality of asthma apps, from the perspective of both asthma self-management education and behavior change strategies. The framework features 10 dimensions and corresponding items, which reflect the material content of asthma apps currently available for download on smartphones. This framework is an important first step in using asthma apps as part of the set of strategies available to healthcare providers to improve quality of life (QOL) among asthmatics.

Through the three-round Delphi survey process, the number of items to be included in the assessment framework was reduced from 87 to 62, by merging overlapping items and deleting items that would be difficult to operationalize and measure, based on feedback. Asthma self-management should address asthma knowledge, skills training for effective self-management, non-pharmacological interventions, goals and planning, feedback and monitoring, shaping knowledge, social support, and prompts (i.e., brief messages that encourage the user to engage in particular behaviors).

Among the dimensions, *skills training for effective self-management* had the highest weight (0.203), followed by *asthma knowledge* (0.105), *shaping knowledge* (0.105), *ease of use* (0.105) and *usability* (0.105). Therefore, *skills training for effective self-management* is the most important factor in asthma self-management, from the perspective of participating experts and consistent with the literature [50, 51]. Moreover, reports of web-based interventions have shown that interventions involving more behavior change techniques are indeed effective [52].

The framework can be used to create an evaluation instrument which could then be tried out and evaluated itself for validity and reliability.

### Limitations

This study identified a framework and a needed next step would be to derive and validate an actual instrument. The framework only reflects the judgement of the participants’ choosen and that another group, perhaps in another country or composed of more multidisciplinary experts, might produce a different framework. The fact that participants were all asthma experts explains why the framework’s content is so heavily focused on the disease and its treatment and why an essential item for all health related apps like privacy and security is missing. Cost, software reliability, and whether patients understand the information apps present might be concerns of those in the telemedicine field. While ease of use may touch upon this, another telemedicine concern flowing from understandability is how much knowledge apps assume patients have.

In addition, the whole research was conducted in China (a middle-income Asian nation). The sample size was small, leading to many semi-qualitative results. Also, the path for future research in applying the framework in culturally diverging regions of lower (e.g. sub-Saharan Africa) and/or higher (e.g. Europe) exists. The framework designed surveyed providers (and not patients) about what they think is good for patients. Future research might include an analogous methodology used with severely affected (“expert”) asthma patients, etc. Still, the current framework provides guidance for assessing asthma content and behavioral strategies in existing apps on developing new one.

## Conclusion

This study involved 29 experts who were active in respiratory disease field for more than 5 years. The assessment framework created can be used to develop evaluation instruments for asthma apps that can be used by health researchers and healthcare professionals wishing to incorporate them in their treatment plans and to guide the development of quality asthma apps supporting patient self-management. Among them, portion of behavior change strategies of the framework can be used in evaluation of HIT apps for other chronic and common disorders.

## Data Availability

The dataset supporting the conclusions of this article is not available since the privacy of residents is included.

## Abbreviations

AHP: Analytic hierarchy process
apps: applications
BCT: Behavior change techniques
CI: Consistency index
CNKI: China National Knowledge Infrastructure
CNNIC: China Internet Network Information Center
CR: Consistency ratio
CV: Coefficient of variation
QOL: Quality of life
RCI: Random consistency index
SD: Standard deviation
